# The development and implementation of a blended video watching and peer learning model for master’s nursing students: a quasi-experimental study

**DOI:** 10.1186/s12912-023-01204-0

**Published:** 2023-03-07

**Authors:** Arkers Kwan Ching Wong, Tommy Tsz Man Hung, Jonathan Bayuo, Frances Kam Yuet Wong

**Affiliations:** School of Nursing, The Hong Kong Polytechnic University, 1 Cheong Wan Road City, Hung Hom, Hong Kong

**Keywords:** Education, Nurse practitioners, Nursing, Professional Development, Teaching

## Abstract

**Background:**

With today’s complex needs of the population and high demands in quality of care, there will be a continuing need for expanding role of nurses to assume more responsibilities in healthcare. Newly graduated nurses, who possess the competence to function as Registered Nurses, will soon recognize that lecture-based, passive delivery of content is not sufficient to deal with the complex healthcare environment.

**Aim:**

This study aimed to compare the effects of a blended video watching and peer learning program and the usual lecture-based program on the levels of satisfaction and self-confidence in learning, perceptions of peer learning, and academic performance of students enrolled in a master’s nursing program.

**Methods:**

A quasi-experimental study was conducted. The program was offered to Master of Science in Nursing students during Spring 2021 (intervention group, n = 46), while the usual face-to-face lectures and tutorial classes were provided to students enrolled during Fall 2020 (control group, n = 46).

**Results:**

There was a statistically significant increase in satisfaction, self-confidence in learning, and academic performance in the intervention group after learning in a blended video-watching and peer learning mode.

**Conclusion:**

This study fills a knowledge gap to meet the learning needs of time-conscious, part-time students working full time in hospitals.

## Contributions to the literature

- This paper equips nurses with advanced knowledge and competence in solving complex clinical situations and eventually prepare them to progress their careers to the advanced practice nurse level.
- TA blended video watching, and peer learning model will provide evidence support to further develop online learning and peer teaching that suits the learning needs of mature students.

## Background

Although in recent years online, interactive learning formats are increasingly being adopted, master-level nursing programs have employed face-to-face lectures as their primary channel for delivering knowledge to students [1]. With this lecture-based, didactic method of teaching, students often face difficulties in analyzing, synthesizing, or evaluating what they are taught, and applying the knowledge gained in class to their clinical working environment [2]. Several studies found that the students enrolled in such courses, who were largely working as full-time registered nurses, were not engaged, and motivated enough to actively participate in learning because this passive approach to learning did not support understanding and knowledge retention and stimulate inquiries during the learning process [3, 4]. The complexity and diversity of advanced nursing practice can also make it hard for students to fully comprehend and understand the ways in which they must develop to meet professional expectations, given a one-way communication teaching format [5].

Video watching and peer learning are two educational modalities that promote learning by allowing junior nurses to learn essential knowledge and skills at their own time and pace. Video learning is particularly valuable to master’s nursing students who are working shifts since the videos can be made conveniently available on Internet-based video-sharing sites, thereby eliminating geographic barriers, and providing time and cost savings. Studies have revealed that using a video as a teaching medium can increase the students critical thinking abilities [6] and help them to improve and retain their levels of knowledge and competence [7]. There is also evidence to suggest that such an approach is comparable to face-to-face lecture sessions in terms of student satisfaction level [8]. A Turkish study showed that a video-based learning program, which was comprised of a 2-hour Powerpoint presentation and simulation regarding advanced care techniques for newborns, improved the students’ academic and practical performance, as evaluated on the basis of their objective structured clinical examination scores [9]. Similarly, studies have reported improved examination scores and student satisfaction among nursing students following the implementation of video-based learning [10, 11]. Beyond nursing students, another study observed that video-based learning was effective in preparing physiotherapy students for practical examinations because it led to reduced anxiety and improved academic performance [12]. Despite the notable benefits, some studies have highlighted challenges associated with video-based learning. In the phenomenological study that explored student experiences with video-based learning in Iran, the authors observed that some content were out of sync with the context coupled with time pressures for students to complete the programme [13]. Another mixed method study also observed that the learning climate affected how well the students navigated the blended learning platform [14].

Peer learning is another teaching pedagogy that is used to motivate students to actively participate in constructing their own concept and understanding based on their previous knowledge. Peer learning refers to the building of new knowledge through active learning support from peers [15]. Peers can help their fellow students to readily integrate new knowledge and innovative ideas and learning experiences into the curricular context [16] and are deemed by students to be more approachable than subject lecturers. Learning from peers is particularly important for novice or junior nurses since they can receive information about the different nursing cultures and practices from colleagues who work in diverse clinical practice settings, thus allowing them to reflect on and improve their own practice early in their career [17]. A systematic review that examined the value of peer learning among undergraduate nursing students underscored that it was associated with an improvement in self-confidence and competency, and a decrease in anxiety [18]. In another qualitative meta-synthesis, which included six qualitative studies, it was observed that peer learning contributed to the learning process of undergraduate nursing students in preparing them to become professional nurses through personal and professional development [19]. Similar findings were resonated in a recent qualitative study that sought to examine preceptors and nursing students’ experiences of peer learning in a mental health context [20]. The authors noted that the process of peer learning facilitated the establishment of a supportive relationship and reciprocal learning which enabled knowledge and skills acquisition [20]. These positive findings notwithstanding, the potential benefits of peer learning among postgraduate nursing students remain poorly articulated in existing literature as the studies have significantly focused on undergraduate nursing students.

By combining video watching and peer learning activities, nurses have the freedom to prepare learning activities outside the set classroom time, which allows them some flexibility to engage in self-directed learning, reflection, and critical thinking. Instead of emphasizing one-way lecturing and face-to-face contact hours, the focus is on devoting more time to collaborative learning. All students can benefit from learning with and from each other. Incorporating these two concepts may be challenging due to logistic and timetabling issues, yet it is worth making the effort due to the above-mentioned benefits.

Considering the individual positive effects of video-based and peer learning, what their combined effect might be for nursing students is a matter of interest. There is a dearth of studies combining both video watching and peer learning as a teaching methodology. One recent study analyzed the use of self- and peer-assessments of a video of a group of physician assistant students practicing their clinical skills. It was found that the students increased their awareness of their own strengths and weaknesses in verbal and nonverbal communication and increased their motivation to practice [21]. Similar positive results were found among nursing students on pre- and post-operative care knowledge [22], among medical students on learning satisfaction [15], and among physiotherapists on clinical performance [23]. To the best of our knowledge, the proposed active-learning strategies have not been attempted in a post-baccalaureate nursing program. Given the many potential positive attributes of the proposed approach, an attempt is made here to develop and compare the effectiveness of a blended video watching and peer learning program and the usual lecture-based program on the satisfaction and self-confidence in learning of Registered Nurses enrolled in a Master’s Nursing program, and on their perceptions of peer learning and their academic performance. The overall aim of this study was to compare the effects of a blended video watching and peer learning program and the usual lecture-based program on the levels of satisfaction and self-confidence in learning, perceptions of peer learning, and academic performance of students enrolled in a master’s nursing program.

### Methods

#### Design and setting

The present study adopted a quasi-experimental, non-equivalent control group design. It was conducted at a university in Hong Kong. The study has been reported according to the Transparent Reporting of Evaluations with Nonrandomized Designs (TREND) checklist [24].

### Sample and assignment method

Students enrolled in the subject “Concepts of Advanced Nursing Practice” in a Master of Science in Nursing program at the university during the Fall 2020 or Spring 2021 semester were recruited to participate in the study. Only postgraduate students enrolled in the specified course, working in any healthcare facility in Hong Kong, and willing to participate were considered eligible to participate. Students who were enrolled during the Fall 2020 semester and who agreed to participate were recruited to the control group, while those enrolled during Spring 2021 were recruited to the intervention group with their consent. The priori sample size was calculated by using Raosoft® software. Assume a margin of error of 10% and consider a confidence interval of 95%, the total sample size required was 92 participants. Both groups were taught by the same teaching team, consisting of one Chair Professor, one Assistant Professor, and three Advanced Nurse Practitioners from the Hospital Authority (HA).

### Conventional teaching approach (control group)

Conventionally, the learning activities of this subject employed lectures as the primary channel to deliver theoretical knowledge. One Professor and one Assistant Professor provided the theoretical and evidence-based input of the subject titled “Concepts of Advanced Nursing Practice” in five lectures, with three advanced nurse practitioners from the Hospital Authority providing lectures illustrating how advanced nursing practice is realized in their day-to-day work. Apart from the lectures, three face-to-face 3-hour tutorial sessions were held as a platform for stimulating ideas and generating debate on key concepts of advanced practice in nursing, which included the seven domains of the advanced nursing practice competence framework. In these tutorial sessions, students were divided into small groups to engage in discussions with the subject lecturers on the topic of the seven domains of the advanced nursing practice competence framework formulated by the Hong Kong Academy of Nursing [25]. Students were required to show their understanding and to provide examples of how an Advanced Nurse Practitioner in a selected clinical area can fulfil some or all these seven domains.

### Blended video watching and peer learning program (intervention group)

Instead of face-to-face tutoring, the three Advanced Nurse Practitioners (APNs) who worked in the three specialty areas of critical care, wound care, and midwifery, recorded, and uploaded their daily work on an online blackboard platform. The Chair Professor and Assistant Professor in this study provided theoretical input alongside the recorded content by the APNs and moderated the online platform during discussions or question/answer sessions. The contents of the video included comprehensive health assessments, relationship building, physical examinations and history taking, patient education, and advanced nursing management which were released to the blackboard at least a week to the scheduled module date. It is worth mentioning that the content of the videos was congruent with the subject matter of concepts of Advanced Practice Nursing. The videos lasted approximately 10 min with an option to repeat each viewing if required. The purpose of providing videos illustrating the work of Nurse Practitioners in clinical settings was to give the students an idea of advanced nursing practice and facilitate their discussion online. The Advanced Nurse Practitioners were present in the online discussions that followed afterwards to respond to questions and engage in discussions with the students about the advanced nursing practice in their real-life settings. These online discussions were held on the blackboard platform. No time limits were set for these discussions, and they depended on the questions that the students had noted. The lead author of the study served as a moderator for all the sessions.

In addition to watching the videos, peer learning activities were provided to students on an online platform. Students were randomly organized in constant groups of 5–6 during all three virtual tutorial sessions. Before the sessions, one or two members of each group were required to use their own time to interview an Advanced Nursing Practitioner in their own working environment to compare the similarities and differences of levels of competencies of the work of advanced nursing practitioners in the video. These tutorial sessions were moderated by the Assistant Professor. This comparison stimulated the students to probe further how advanced nursing practice can happen in reality taking into account of the factors of contextual and professional factors. In the first session, each group had to write a simulation case scenario based on what they had learned in the videos. The content of the simulation case scenario had to include the background, medical history, chief complaint, progress, investigation results, and medical treatments of a simulated patient based on the students’ area of specialty. In addition, they had to think of some nursing measures that could fulfill at least three of the seven competence domains of the advanced nursing practice competence framework. In the second session, they had to pass the simulation case scenario description to another group, and in return receive that group’s simulation case scenario. In this second tutorial session, students had to hold a discussion with their groupmates and use the knowledge gained from the lectures, videos, and formulate a nursing care plan that could suit at least three of the seven advanced nursing practice competence domains and the simulated patient situation. In the third tutorial session, the group that wrote the simulation case scenario would take turns providing guidance and feedback to the group that formulated the nursing care plan in accordance with the scenario description. The aim of this final session was to allow both groups to reflect upon, discuss, and clarify issues relating to the patient clinical presentation and the care of the Advanced Nurse Practitioner, and to communicate and exchange experiences within and across the groups. The virtual discussion platform allowed students to learn from each other by sharing their experiences related to their own work environment without the need to travel to school. This peer learning arrangement augmented group learning effects because each member in the group bring his/her unique learning experiences to share with other group members. The discussion forum provided a new way of gathering the students together and implementing peer support where the students were able to reflect their learning experiences in a safe environment. Compared to the conventional face-to-face discussion that was led by the subject lecturers in tutorial class, this discussion forum among peers could promote critical thinking and reasoning skills, use their own experience to enrich each other’s learning.

After the three virtual tutorial sessions, each group was required to create a video that mimic the videos made by the advanced nursing practitioners, using the outcomes of the third tutorial session as a script. In the video, some students acted as patients and one student acted as an advanced nursing practitioner who provided advanced nursing care. The dialogue and interactions between the patients and the advanced nursing practitioner were based on the comments and feedbacks from the peers in virtual tutorials. Student-generated video, used as one of the assignment components of this subject, could reflect whether the students were able to fulfill the learning outcomes such as identifying issues encountered by the advanced practicing nurse in their daily work, understanding the roles and responsibilities of advanced nursing practitioners in clinic, and indicating the differences between a registered nurse and an advanced nursing practitioner.

All the students were present for all the peer learning activities with no absentees recorded. As with the control group, students in the intervention group presented their critical analysis of the advanced nursing roles in these competence domains substantiated with literature and the use of a real clinical case in the format of a group presentation after the three tutorial sessions.

### Data collection

For each cohort of students, data were collected at two time points, pre- and post-intervention. After the students had been divided into groups in the first lecture, a project assistant who was not involved in teaching and blinded to the student groupings was responsible for providing and collecting the questionnaires. The project assistant was trained and five per cent of the gathered data were randomly selected for an independent review to ensure the quality of the data. Following recruitment, the project assistant contacted the participant to complete a bundle of questionnaire. Following this, the project assistant reviewed it for completeness. Following the completion of the intervention, the same procedure was followed to complete the questionnaires.

### Instruments

There were four outcomes evaluated in this study: satisfaction, self-confidence in learning, perceptions of peer learning, and academic performance in the advance nursing practice course. There were five sets of measures, including a background demographic survey, the Student Satisfaction and Self-confidence in Learning scale, the perceptions of peer-assisted learning questionnaire, a rating scale for video content and quality, and an assessment criterion for measuring the performance of students in illustrating their understanding of the seven competence domains of advanced nursing practice.

The data collected in the background demographic survey included age, gender, marital status, hospital type, years of working as a registered nurse, and department in which one worked.

The Student Satisfaction and Self-confidence in Learning scale has two subscales, satisfaction, and self-confidence, with a total of 12 items [26]. The tool was used to assess student satisfaction and self-confidence in learning. The satisfaction subscale contains five assessment items, and the self-confidence subscale has seven items. Both are measured using a five-item Likert scale, with higher scores represent higher satisfaction and greater levels of self-confidence [26]. The scale has high internal consistency, with a Cronbach’s alpha of 0.94 for satisfaction and 0.87 for self-confidence [27].

The perception of peer-assisted learning questionnaire was developed by Sevenhuysen and her colleagues [28] and Anantharaman et al. [29]. This 10-item questionnaire was used to measure a student’s perception of using peer learning as a teaching method. Each item is rated on a 5-level scale from 1 (strongly disagree) to 5 (strongly agree). The total score ranges from 10 to 50, with a higher score indicating a better perception of peer learning.

The content and quality of the videos uploaded by the three APNs were evaluated by the students using a 5-item scale. The scale measures the overall quality of the video, its technical quality, fluidity, loading, and the general experience of the students after watching the video. Students rated each item with 1 = very inappropriate to 5 = very appropriate, with higher scores representing the perception that the videos were of better content and quality. The face validity and content validity (CVI = 94%) of the scale were established.

For academic performance in relation to the subject, students were required to make a 7-minute video that demonstrated their understanding of the concepts of advanced nursing practice using seven domains of advanced practice nursing competence. The videos were assessed according to a set of criteria that included the students’ ability to collect and process information, identify, and prioritize relevant issues or problems, implement intervention strategies, evaluate outcomes, and make required modifications, as well as the students’ self-reflections. The total score for this assignment was 100. The face validity and content validity (CVI = 91%) confirmed the criteria was validated.

### Data analysis

IBM SPSS, Version 26.0 software was employed for data analysis in this study. Descriptive analyses were used to describe the data collected at baseline, such as the background demographic data of the students. All the demographic data of the students in the two cohorts were compared to ensure that there were no significant differences between the groups and to determine if there was any need to adjust through a sensitivity analysis.

Perceptions of Peer Learning and the rating of the content and quality of the video were presented in mean and standard deviations. An independent sample t-test or a Mann-Whitney U test was used to compare differences in the demographic data, in Student Satisfaction and Self-confidence in Learning, and in the performance of the two cohorts. A Paired t-test or Wilcoxon signed-rank test was adopted to compare pre- and post-intervention differences in Student Satisfaction and Self-confidence in Learning for both cohorts. The Cohen’s d was also calculated from the group means, as well as the pooled standard deviation, to ascertain the effect sizes following the implementation of the teaching pedagogies. Per-protocol was used for missing data.

### Ethical consideration

The University’s institutional review board approved the study. Information about and an explanation of the ethical considerations of the study were provided to all the students, and they had to sign a consent form prior to the commencement of the study. They were also reminded that their personal information and responses would be kept confidential.

## Results

### Participant flow and sample characteristics

A total of 120 participants were screened for eligibility. Following screening, 92 participants met the criterion for inclusion and agreed to participate. These participants were assigned to the two arms based on the subject enrollment period, with 46 in both the intervention and control groups. The response rates were high for both groups, with 77% (46/60) of students in each semester agreeing to participate. The mean age of the students was 29 years. Most of the participants were single and working in a public hospital. Of the 92 students, more than half had been working as a registered nurse for 1–3 years, a quarter had been working for 4–6 years, and around 15% had been working for more than 7 years (Table 1).

**Table 1:** Demographic characteristics of participants

		Total (n = 92)		Intervention group (n = 46)		Control group (n = 46)		
		Count	Table Valid N %	Count	Column Valid N %	Count	Column Valid N %	*p*-value
Gender	Male	20	21.7%	11	23.9%	9	19.6%	0.80^a^
	Female	72	78.3%	35	76.1%	37	80.4%	
Age	Mean (SD)	28.8	(4.64)	29.2	(4.91)	28.4	(4.37)	0.42^c^
Marital status	Single	76	83.5%	36	80.0%	40	87.0%	0.41^b^
	Married	15	16.5%	9	20.0%	6	13.0%	
Work setting	Private hospital	11	12.0%	7	15.2%	4	8.70%	0.52^b^
	Public hospital	81	88.0%	39	84.8%	42	91.3%	
Work experience	1–3 years	55	59.8%	28	60.9%	27	58.7%	0.74^a^
	4–6 years	23	25.0%	10	21.7%	13	28.3%	
	7–9 years	13	15.2%	7	17.4%	6	13.0%	

Note:*SD* = *Standard deviation*; a = Pearson Chi-square test; b = Fisher’s exact test; c = Independent t-test; *p < .05

### Student satisfaction and self-confidence in learning

The Student Satisfaction and Self-confidence in Learning scores for participants in both the intervention and control group participants are summarized in Table 2. The findings suggest that while students in both groups showed an increase in satisfaction and self-confidence post-intervention when compared to pre-intervention, only the intervention group showed a statistically significant difference in their total score for student satisfaction and self-confidence in learning (*p* = .048), and in their self-confidence in learning sub-score (*p* = .049). There was a small effect size for the intervention on improving student satisfaction (0.39) and student self-confidence (0.40). The overall effect size for the entire scale remained small, yet positive (0.42), highlighting the potentially positive effects of video-based and peer learning.

**Table 2:** Comparison of the effect of blended video watching and peer learning and conventional teaching approach

		n	Pre-intervention Mean (SD)	Post-intervention Mean (SD)	*p*-value
Student satisfaction and self-confidence in learning (Score range: 12–60)					
Control	46		44.7 (6.07)	45.8 (5.70)	0.160
Intervention	46		46.0 (5.83)	48.4 (6.53)	0.048*
*p*-value			0.34	0.073	
Cohen’s *d*				0.42	
Student satisfaction sub-score (Score range: 5–25)					
Control	46		18.5 (3.27)	19.1 (3.04)	0.58
Intervention	46		19.4 (3.24)	20.4 (3.63)	0.083
*p*-value			0.22	0.089	
Cohen’s *d*				0.39	
Self-confidence in learning sub-score (Score range: 7–35)					
Control	46		26.2 (3.19)	26.7 (2.96)	0.52
Intervention	46		26.6 (3.10)	28.0 (3.51)	0.049*
*p*-value			0.56	0.10	
Cohen’s *d*				0.40	
Academic performance (Score range: 0-100)					
Control	46		/	71.7 (4.79)	/
Intervention	46		/	74.0 (2.61)	/
*p*-value			/	0.004*	
Cohen’s *d*				0.60	

Note: *Statistically significant as it is < 0.05

### Academic performance

Table 2 shows the academic performance of the participants in both the intervention and control groups. When compared to the control group, the intervention group had statistically significant higher scores in this subject (*p* = .004). The Cohen’s d that was calculated based on the group means and pooled standard deviation showed a positive, medium effect size (0.60).

### Video content and quality ratings

Students in the intervention group were required to watch the videos taken by the three advanced nurse practitioners and rate them in seven dimensions: overall quality, technical quality, fluidity, loading, experience, length, and content (Table 3). In summary, students enjoyed watching the videos and accorded the highest score to the overall quality of the video (x̄ = 3.85, SD = 0.69), then to its technical quality (x̄ = 3.78, SD = 0.76), and then to its contents (x̄ = 3.73, SD = 0.81). Fluidity (x̄ = 3.66, SD = 0.76), loading (x̄ = 3.66, SD = 0.86), and length of the video (x̄ = 3.66, SD = 0.88) were scored comparatively lower.

**Table 3:** Rating for video content and quality (N = 46)

Items	Questions	Mean (SD) score on each dimension (1 = very poor, 2 = below average, 3 = average, 4 = above average, 5 = very good)
The overall quality of the video	How would you evaluate the overall quality of the video?	3.85 (0.69)
Technical quality	How would you evaluate the technical quality of the video in general?	3.78 (0.76)
Fluidity	How would you evaluate the fluidity of the video playback?	3.66 (0.76)
Loading	How would you evaluate the loading time of the video?	3.66 (0.86)
Experience	How would you evaluate your general experience during video playback?	3.68 (0.76)
The length of video	Do you think the length of the video is appropriate?	3.66 (0.88)
The Content of the video	How would you evaluate the content of the video?	3.73 (0.81)
Total (range: 7–35, higher scores representing better perceptions of video content and quality)		26.0 (1.98)

### Perceptions of peer learning

After completing the program in the Spring 2021 semester, students in the intervention group expressed their views on peer learning in the Perceptions of peer-assisted learning questionnaire. As seen in Table 4, students indicated overall satisfaction with this new type of learning approach. They agreed that their communication with their peers was good (x̄ = 3.93, SD = 0.61). They found it easy to receive (x̄ = 3.90, SD = 0.63) and deliver (x̄ = 3.85, SD = 0.65) feedback from and to their peers, and they thought that peer learning covered concepts that were appropriate for their knowledge and experience (x̄ = 3.85, SD = 0.57). Recommending the same method for other postgraduate nursing students (x̄ = 3.66, SD = 0.83) and the notion that peer-assisted learning helped to retain new concepts and knowledge better than lecture teaching however scored relatively lower (x̄ = 3.71, SD = 0.75).

**Table 4:** Perceptions of peer learning (N = 46)

Items	Mean (SD) (1 = strongly disagree, 2 = disagree, 3 = no comment, 4 = agree, 5 = strongly agree)
The peer-assisted learning covered concepts that were appropriate for my knowledge and experience	3.85 (0.57)
I found it easy to receive feedback from my peer mentor	3.90 (0.63)
I found it easy to deliver feedback to my peer mentor	3.85 (0.65)
I found the peer-assisted learning more interactive than lecture teaching	3.83 (0.63)
The peer-assisted learning has helped you in presentation preparation	3.80 (0.72)
Peer-assisted learning has improved my understanding of the role and responsibilities of APN	3.80 (0.81)
Communication with peer mentor was good	3.93 (0.61)
Peer-assisted learning helped me retain new concepts and knowledge better than lecture teaching	3.71 (0.75)
You recommend the same method for other postgraduate nursing subjects	3.66 (0.83)
Overall, learning with peer mentor in this format was a positive experience	3.78 (0.69)
Total (range: 10–50, higher scores representing better peer learning perceptions)	38.1 (6.05)

## Discussion

The current study demonstrated that students in the intervention group experienced a significant increase in their overall satisfaction and self-confidence in learning, and in their academic performance after learning in a blended video-watching and peer learning mode. In addition, when compared to pre-intervention, the post-intervention increases in the sub-scores for student satisfaction and self-confidence in both the control and intervention groups hints at the educational value of both the conventional teaching approach and the blended approach.

Past teaching strategies that focused on the lecture-based, one-way passive delivery of content can no longer satisfy the learning requirements of students today [30]. Besides, the emergence of the Coronavirus pandemic creates room to consider innovative, blended approach to teaching and learning. In this program, the three APNs transformed their lecture content into videos of simulated practice to illustrate their daily work. The students, for their part, acted as both peer tutors and students in the subject. The learning activity required the students to teach each other and provide feedback in an online tutorial setting to deepen their understanding and improve retention in a topic that they had prepared for and delivered. This concept of pedagogy is consistent with social constructivist theory [31]. Social constructivist theory suggests that learning occurs in a social context where interactions, relations, participation, and communication with peers happen in a shared environment [32]. The high confidence in learning scores post-intervention confirmed that this active learning strategy can facilitate students to engage in the processes of critical thinking and reasoning. Students who are satisfied in their learning experiences may remain engaged, motivated and feel prepared to face learning challenges [31]. As noted in previous studies [19, 20], peer learning is helpful in facilitating learning and knowledge acquisition as it offered the opportunity to learn from one’s colleagues. The current study offers some evidence that the benefits of peer learning observed among undergraduate nursing students may be present among postgraduate nursing students.

The videos used in this study served as a good medium for teaching, as evidenced by the high score ratings from the students. In fact, it is a commonly used method in lecture settings to help nursing students grasp theoretical knowledge on advanced nursing practices [33]. However, the reality of what the Nurse Practitioner does in a nurse-led clinic is far more difficult to learn and understand without clinical visits. The videos produced by our Nurse Practitioners and film-making company have a visual impact as they show our students how Nurse Practitioners use gestures and facial expressions to interact and communicate with patients and demonstrate some advanced skills such as the performing of physical examinations, taking of patient histories, nursing management, and counselling. Different from group viewing situations in classroom or lecture settings, students can see everything from the use of verbal and nonverbal communication to the finely tuned skills of the Nurse Practitioner, in front of a monitor without anyone blocking their view.

The videos, unlike the conventional face-to-face approach offers the unique opportunity of revisiting them and learning as much as one wants to. This approach therefore may have offered some flexibility considering that the participants are practicing nurses faced with varying time pressures. Previous studies have also showed that the pause and rewind function of video-taped contents allowed students to engage in self-paced learning [34, 35]. Bloomfield and Jones [36] found that by revisiting video-taped contents graduate first-year pre-registration nursing students enhanced their acquisition of clinical skills. Bas-Sarmiento et al. [37] adopted videos as an essential component in training nursing students in non-verbal communication, establishing a helping relationship, and promoting an empathetic nurse-patient relationship.

### Limitations

There are several limitations to this study. First, blinding of the three advanced nurse practitioners was not feasible, which may have led to the differences in academic performance scores between the intervention and control groups. However, the subject has a stringent assessment rubric for Nurse Practitioners to follow to minimize possible biases. Second, the number of students recruited to this study was relatively small, and the study was conducted at one institution. The findings of the study may therefore not be generalizable to nursing students enrolled in master’s programs in other universities. Finally, a qualitative enquiry with both the students and Nurse Practitioners was not performed, leading to difficulty in achieving an in-depth understanding of the results of the current study.

## Conclusion

This study indicated that the blended video watching, and peer learning program may be an effective pedagogical strategy for increasing the self-confidence and satisfaction in learning, and academic performance of master’s students. Because of the positive perceptions of peer learning, we recommend that active learning approaches, emphasizing self-learning, and co-responsibility in learning, be adopted in master’s programs. Further randomized controlled trials adopting the double-blinded approach would be needed to confirm these results.

## Data Availability

The datasets generated and/or analysed during the current study are not publicly available due to a large dataset but are available from the corresponding author on reasonable request.
